# Miniaturisation of the *Daphnia magna* immobilisation assay for the reliable testing of low volume samples

**DOI:** 10.14324/111.444/ucloe.3037

**Published:** 2025-01-29

**Authors:** Eberhard Küster, George Gyan Addo, Silke Aulhorn, Dana Kühnel

**Affiliations:** 1 Department Ecotoxicology (former Department Bioanalytical Ecotoxicology), Helmholtz Centre for Environmental Research – UFZ, Leipzig, Germany

**Keywords:** miniaturisation, extract testing, leachate testing, microplastic, nanoparticle, environmental monitoring, groundwater, crustacea, pesticide, plankton testing

## Abstract

International standard test guidelines for the ecotoxicological characterisation of various substances use organisms such as algae, daphnids and fish embryos. These guidelines recommend or use relatively high volumes of water for the process of testing, for example, 200 mL for a complete dose–response relationship in a daphnia assay. However, for various samples such as concentrated extracts from environmental monitoring or leachates from microplastic ageing experiments, the amount of available sample volume is limited, that is, rather in the range of 10–50 mL/biotest. Using the exposure volumes as recommended in test guidelines would not allow to test a range of different concentrations or to repeat tests or use multiple different organismic bioassays. Lower media volumes would allow the testing of more samples (more concentrations per sample, more test repetitions for statistical robustness, etc.) but it may also decrease the possible number of organisms tested in the same volume. Here, we aimed at reducing the test volumes in the acute daphnia assay (using a maximum of 30 mL for a complete dose–response relationship) without impacting animals’ sensitivity towards toxicants. A literature review on existing miniaturisation approaches was used as a starting point. Subsequently, assays employing conventional as well as reduced test volumes were compared for 16 selected test substances with a diverse spectrum of lipophilicity. Results showed that there are differences in EC_50_ between the two approaches, but that these differences were overall only within a range of a factor of two to three. Further, by retrieving EC_50_ values for the genus *Daphnia* and 16 test substances from the United States Environmental Protection Agency database, we demonstrated that our results are well in line with the general differences in sensitivities.

## Introduction

Most guidelines for aquatic ecotoxicity testing were established for the testing of individual substances, usually not restricted regarding their availability. For example, the International Organization for Standardization (ISO) [1] (6341:2012) and Organisation for Economic Co-operation and Development (OECD) [2] TG 202 (describing the acute *Daphnia* test over a duration of 48 h) recommend test volumes of 2 mL per daphnia neonate, adding up to a recommended 10 mL per test concentration when testing five neonates per technical replicate. This adds up to a total volume of 50 mL for the generation of a complete dose–response relationship with five test concentrations if only a single experiment is done and only one technical replicate is used (controls not included here). With usually using four technical replicates per test concentration or dilution, this further increases the needed volume to 200 mL in a single experiment. As a consequence, volumes and replicates needed for the testing are normally very large as restriction of volume or numbers of replicate tests is not an obstacle with single substance testing. These volumes, as well as the number of neonates per replicate, are seen as prerogative in terms of the robustness of data sets and subsequent statistical reliability which is needed for the hazard evaluation of single substances. If one needed to do tests with other standard organisms, such as algae or fish embryo, the required sample volumes would increase even more.

However, in several cases there is a restriction on sample volumes, for example, in ecotoxicological monitoring of environmental water samples. Currently, European ground- and surface-water monitoring focuses on chemical analyses (European Water Framework Directory [WFD] and its daughter regulations). As usual not all substances in an environmental sample are analysed (which is obviously not possible). But in an environmental sample the mixture of all substances – and not only a few substances of interest – contributes to overall ecotoxicity. Thus, applying ecotoxicological tests is seen as a valuable complemental method to chemical analysis, making it possible to include and evaluate the overall toxicity of all bioavailable substances in a mixture [3–5]. At the same time, the sample volumes for the different tests are often restricted, as obtaining and preparing water samples for monitoring is elaborate and costly. Chemical analysis usually requires sample volumes in the μL to mL range. In contrast to that, as demonstrated above, ecotoxicological tests with organisms often need sample volumes above 200 mL per sample. This may be one reason why ecotoxicological tests such as the acute *Daphnia* immobilisation assay are not as often used as might be helpful. The same restrictions apply to other types of samples such as leachates prepared from microplastics [6,7], fractionated microplastics samples and other materials with limited sample availability [8,9].

Standard tests with daphnids carried out in our laboratory have so far used 15 mL of medium for five neonates (one neonate per 3 mL) and four technical replicates adding up to 60 mL for a single concentration (e.g., [10–12]). This is in the following referred to as the ‘conventional approach’. A dose–response curve with a 1:2 dilution thus needs 120 mL of sample volume for a single experimental run.

This motivated this study, which aimed at developing a robust but sensitive *Daphnia magna* immobilisation test requiring less sample volume than the conventional assay.

We used the review by Grintzalis et al. [13] as a starting point for our miniaturisation approach and complemented this database by using additional approaches retrieved from the scientific literature (e.g., [14,15]). This first literature screening indicated three basic approaches to achieve a reduction in sample volume for the miniaturisation of the *Daphnia* assay: reduce the ratio volume-per-neonate (i.e., increase density), reduce the number of concentrations tested or reduce the amount of neonates per replicate or concentration. In addition, the impact of miniaturisation on animal fitness and behaviour was studied. Based on this review a scheme for a miniaturised *Daphnia* assay in a 24-well format was developed. In the following, this approach is referred to as the ‘miniaturised approach’ [15]. As a goal we wanted to demonstrate that under miniaturised test conditions no changes occurred in terms of the EC_50_ values of the respective substances, and that factors such as increased animal density would not impact the sensitivity of the test organisms. This was done by comparing the conventional approach to the miniaturised approach by testing 16 selected chemicals. Substance selection was based on lipophilicity as well as test data availability for the conventional approach. Finally, our results were put into the context of general sensitivity differences by comparing them to results obtained with the genus *Daphnia* and the 16 test substances. This was done by retrieving the respective EC_50_ values from the US EPA ECOTOX database.

## Materials and methods

### Literature review

A literature search for existing miniaturisation approaches for the *Daphnia* immobilisation assay (keywords: daphnia AND miniatur*) in the Web of Science™ database was done, based on the PRISMA (Preferred Reporting Items for Systematic reviews and Meta-Analyses) guidance paper [16]. In addition, the so-called Abstract Sifter [17] was used with the ‘query run: daphnia magna’ and the follow-up sifter terms ‘miniaturi’, ‘volume’ and ‘well’ to scan the PubMed database. Bibliometric software Zotero (https://www.zotero.org) was used to find and delete the overlap of both databases. The different miniaturisation approaches were compared with the OECD or ISO standard guidelines especially in relation to sensitivity to positive controls and assay parameters such as used volume or density of neonates (summarised in Table 1). This guided in the development of the miniaturisation approach regarding medium volume and animal density.

**Table 1.** Overview of *Daphnia* test miniaturisation approaches reported in the literature and the respective volumes and formats that were adopted or compared

Volume in technical replicate (mL)	Neonates/technical replicate	Density (neonates/mL)	Technical replicates/sample*	Neonates/sample* (or concentration replicate)	Total vol. needed/sample*	Format and material of test container	Reference
10	5	0.5	4	20	40	Chemically inert material (no specific format recommended)	[1,2]
15	5	0.33	4	20	60	15 mL Pyrex glass vial	[10–12]
1.5	5	3.33	2–4	10–20	3–6	24-well (glass)	This study
10	5	0.5	4	20	40	Glass beaker	[15]
10	5	0.5	4	20	40	6-well (PS)	
2	1	0.5	10	10	20	24-well (PS)	
10	5	0.5	4	20	40	6-well (PS)	[18]
2	1	0.5	10	10	20	24-well (PS)	
200	20	0.1	4	80	800	Glass beaker	[13]
12	10	0.83	4	40	48	Glass beaker	
12	10	0.83	4	40	48	Petri dish	
8–12	8–20	0.66–2.5	4	32–80	48	6-well (PS)	
6	10	1.66	4	40	24	12-well (PS)	
3	18	6	4	72	12	24-well (PS)	
1	3	3	4	12	4	48-well (PS)	
0.3	1	3.3	4		1.2	96 well (PS)	
1	1	1	20	20	20	48-well titre plates (PS)	[14]
10	5	0.5	2	10	>20	12 mL plastic (PS?) cell wells	[19]
1	5	5	4	20	4	24-well (PS)^c^	[20]
10	5	0.5	>1^b^	20	40	24-well (PS)	DaphtoxKit F Benchprotocol (Microbiotests Inc.)
0.2	1	5^c^	3–4			24-well^a^ (PS)	[21]
10	5?	0.5^c^	3–4	20	200	Glass tubes	
20	10?	0.5^c^	3–4	40	80	Glass beakers	

All volumes: mL, PS: polystyrene.

*Sample: single concentration in a dose–response relationship or replicate of environmental sample.

^a^Taken from SI table 2 of [21], ^b^as deduced from the bench protocol (downloaded at https://www.microbiotests.com, March 2022) by the company Microbiotests Inc., ^c^deduced from the paper.

### *Daphnia* cultivation and biotesting

The cultivation medium was as described in Klüttgen [22]. Adult daphnids were cultured individually in 80 mL of ADaM (Aachen *Daphnia* Medium, ADaM artificial freshwater) in 100 mL borosilicate Pyrex^®^ glasses (Th. Geyer, Höxter, Germany). Medium was exchanged completely on Mondays and Fridays. Feeding with microalgae (*Scenedesmus vacuolatus*) [23] was adapted to the age of adults and done on Mondays, Wednesdays and Fridays [24]. Daphnids at age of 1, 2 and 3–5 weeks were fed 1 × 10^9^, 2.3 × 10^9^ and 2.7 × 10^9^ fL/animal on Mondays and Wednesdays and 1.5, 3.5 and 4.1 × 10^9^ fL of algae volume on Fridays, respectively. On Fridays, the daphnids were additionally fed with 250 μL brewer yeast (SIGMA, Seelze, Germany) suspension in distilled water (1 g/L). Details of the specific feeding regime are published in the dissertation of Knops [25]. The animals were fed with the equivalent of 0.07 mg carbon/*Daphnia*/day.

After the systematic assessment of existing approaches for miniaturisation (Table 1), we focused on increase in animal density, that is, volume reduction to allow the testing of low volume samples such that we adopted a multi-well-plate format for easier handling and microscopic observation of immobilisation and death of daphnids. Based on this previous considerations, an approach using one-tenth of the regular standard volume of 60 mL was tested and compared to assays conducted with conventional volumes.

Accordingly, the testing was done in (a) 15 mL Pyrex borosilicate vials closed with a lid (i.e. the ‘conventional’ approach), (b) 24-well borosilicate glass well plates (Irlbacher, Schönsee, Germany, 2 mL volume) (i.e., the ‘miniaturised’ approach). The test substances were dissolved and diluted in ADaM. Test substance solution (15 mL or 1.5 mL) was added to each vial or well. Finally, five neonates (<24 h of age) were pipetted using a fixed volume of 50 μL per vial or well. The Pyrex vials were closed with a lid made of polybutylene terephthalate (PBT) screwcaps with inert PTFE-lined rubber discs. The 24-well plates were covered with a self-made glass cover to decrease evaporation. The exposure was done in the dark and at room temperature for 48 h and the daphnids were not fed during the exposure). After 24 and 48 h, immobilised and dead neonates were compared to controls served as the parameter of the toxicity effect. Immobilisation and any other effects were checked using a stereo microscope (Leica Wild MZ-8, Leica, Wetzlar, Germany) during the use of the well plates. Positive (potassium dichromate, p.a., CAS RN 7778-50-9, Fluka Analytics, Seelze, Germany) and negative controls (ADaM medium) were tested in parallel with each substance. For the positive control, usually two alternating concentrations (EC_20_ and EC_50_) of a concentration response curve, which was built up over the last years, were used. The Ph of the dilution with the highest test concentration was measured at the end of the tests. No deviations from the normal were observed. Only with tests of silver nitrate was the test medium pH stabilised to a pH of 7.4 using 30 mM of 3-(*N*-Morpholino)propansulfonsäure (MOPS) buffer. Oxygen content was measured of the highest concentration at several time points. Preliminary tests and results published by [13] did not show any decrease of oxygen over the exposure time.

### Substance selection and data evaluation

For the comparison of conventional and miniaturised approaches, 16 substances were selected (aldicarb, benzyl carbamate, chlorpyrifos, diazinon, dimethoate, erythromycin, methanol [MeOH], metolcarb, phenyl-N,N-dimethyl carbamate, pirimicarb, potassium dichromate, sodium dodecyl sulfate (SDS), silver nitrate, tebuconazole, terbuthylazine and tramadol; see Table 2). Selection criteria included lipophilicity as well as the availability of data for the conventional approach. As the proposed miniaturised system can be seen as an open test chamber the possibility of volatilisation exists. Thus, another criteria checked before testing was the volatility. A Henry’s law constant below 1 Pa m^3^ mol^−1^ is seen as a threshold in this miniaturised system [27]. Only one substance, phenyl-N,N-dimethyl carbamate, had a Henry’s constant above 1 (i.e., 3.5). All other substances constants were in the range 0.001–0.3. For each substance, dose–response curves were modelled using SigmaPlot™ software (version 14, Grafiti LLC, Palo Alto, CA, USA) and EC_50_ values calculated. The EC_50_ values were compared and differences below a factor of 3 were considered to reflect comparable sensitivities of neonates towards the respective substance in both test approaches.

**Table 2.** Toxicity data of the 16 single substances tested in our lab under both the conventional and the miniaturised test protocol (all concentrations are nominal) with 48 h exposure and immobilisation as endpoint

Test substances (alphabetical order) and main usage^e^	CAS RN (MW) logKow	Water solubility^a^ (chem-dashboard) (μmol/L) exp. or predicted median	Baseline tox _Daphnids _48 h (μmol/L)^d^	Geometric mean of daphnid tests collected from the ECOTOX database^c^ (μmol/L) n = number of found and used data	OECD202 standard (this study) EC_50_ (mg/L)^b^ (μmol/L)	Miniaturised test (this study) EC_50_ (mg/L)^b^ (μmol/L)
Aldicarb *Insecticide, Nematicide, acaricide*	116-06-3 (190.26) *1.13*	31,600	1654	1.341 n = 10	0.7 3.679	0.3546 1.864
Benzyl carbamate *Insecticidal and other uses (industrial intermediate product)*	621-84-1 (151.165) *1.20*	447,000	2276	– (no data in ECOTOX db.) n = 0	80–90 562.3	64.17 424.5
Chlorpyrifos *Insecticide, acaricide*	2921-88-2 (350.58) *4.96*	3.19	1.004	0.000697 n = 28	– (not tested)	0.0001301 0.0003711
Diazinon *Insecticide, acaricide*	333-41-5 (304.35) *3.81*	153	11.7	0.0023 n = 37	0.0003–0.0008 0.0051	0.0003645 0.001198
Dimethoate *Insecticide, acaricide*	60-51-5 (229.2) *0.78*	142,000	5871.7	7.7353 n = 12	1.5941 6.955	0.2107 0.9193
Erythromycin *Pharmaceutical/antibiotic*	114-07-8 (733.93) *2.83*	355	181.3	32.700 n = 1	240 327	29.05 39.58
MeOH *Solvent*	67-56-1 (32.04) *−0.77*	31,200,000	84596	– (no data in ECOTOX db.) n = 0	18.26/3.29 569.9/102.7	13.96 435.7
Metolcarb *Insecticide, acaricide*	1129-41-5 (165.079) *1.70*	158,000	812.1	– (no data in ECOTOX db) n = 0	0.06 0.363	0.0343 0.208
Phenyl-N,N-dimethyl carbamate *Insecticide, herbicide, industrial intermediate product*	6969-90-0 (165.079) *1.56*	27,300 (predicted median)	1065.9	– (no data in ECOTOX db) n = 0	4 24.23	1.464 8.868
Pirimicarb *Insecticide*	23103-98-2 (238.29) *1.70*	11,300	1528	0.080 n = 1	0.01013 0.0425	0.005736 0.02407
Potassium dichromate *Oxidising agent, colouring agent and other uses*	7778-50-9 (294.19)	390,910,000	–	1.019 n = 27	1.36 4.623	1.32 4.487
Silver nitrate	7761-88-8 (169.87)	5,860,000	–	0.129n = 25	– (not tested)	0.01174 0.069
SDS *Industrial chemical, surfactant/dispersant*	151-21-3 (288.4)	4610	–, calculated as surfactant: 187.2	33.588 n = 82	5.55 19.244	9.64 33.425
Tebuconazole *Fungicide*	107534-96-3 (307.82) *3.70*	117	11.1	20.52 n = 4 (data from enantiomers included)	7.2798 23.65	13.92 45.22
Terbuthylazine *Herbicide*	5915-41-3 (229.710) *3.21*	37.2	37.8	– (no data in ECOTOX db) n = 0	3.9365 17.137	11.08 48.24
Tramadol *Pharmaceutical, analgetic inhibitor*	27203-92-5 (263.381) *2.63*	1260	63.2	– (no data in ECOTOX db) n = 0	97.8675 371.58	46.99 178.40

^a^Data from Chemistry dashboard (https://comptox.epa.gov/dashboard), experimental data or predicted median.

^b^Complete concentration–effect relationship parameters in the Appendix, Table A1.

^c^Method of data retrieval: see Methods section.

^d^ECOSAR tool (Version 1.11) used within the Epi Suite™ software (neutral organic SAR).

^e^Usage definitions taken from table C of Zenodo data publication in [26] (retrieved 18 July 2024). logKow: from Epi Suite™ (experimental data used, if existing) via https://www.chemspider.com.

### ECOTOX database data retrieval

Beyond the comparison of neonate sensitivities using both the conventional and miniaturised approaches, the general sensitivity of daphnids over various test formats and species, as well as additional variations in tests for the 16 substances, was also assessed. For the calculation of the geometric mean of test results of daphnids exposed to the selected test substances, data from the US EPA ECOTOX database were used. The geometric mean is the metric used to compute species-specific average sensitivity when multiple data are available. Data retrieval from the ECOTOX database was similar for all substances and the following selection criteria were used: habitat → aquatic; chemicals → CAS RN; effect measurements → mortality groups → mortality; endpoints → concentrations based endpoints → LD_50_, LC_50_, EC_50_, ED_50_; species → daphni*; test condition → observation duration (number of days = 2); exposure media → water (salt and fresh); exposure type → only aquatic and static; test location → lab.

## Results

### Literature search

Eleven studies on miniaturisation could be identified with the above-mentioned keywords in the literature database(s) and using the Abstract Sifter [17] specifically using small volumes or microtitre- or well plates of different sizes. Results are summarised in Table 1. The original Abstract Sifter file included 3801 publications dating back to the year 1926 (keyword query run ‘daphnia magna’). Usage of three sifter terms gave 10 publications with a similar frequency count above 4. A screen shot of the first 42 publications found with the Abstract Sifter can be seen in the Appendix (Table A2). Data show that in comparison to OECD and ISO guidelines (the conventional approach) the range of different parameters sometimes cover three orders of magnitude, that is, the volume needed per replicate ranges from 200 μL to 200 mL (mean of 9 mL). The animal density (neonates/mL) covers a little more than one order of magnitude (ranging from one neonate per 0.1 to 6 mL and a mean of 1.5), as does the number of neonates needed per sample (ranging from 10 to 80 animals, mean 18). Regarding *Daphnia* sensitivity, no major differences were observed, and no minimal requirements regarding volume or water column height were made. Accordingly, the approach for miniaturisation tested here would be in the lower range of volume/replicate, neonates and volume per sample needed but would be in the upper range of the animal density (3.3 animals/mL). From the density point of view, it is equal to a single neonate/0.3 mL as it was used by Grintzalis et al. [13] in a 96-well plate. Irrespective of the different volumes, neonate numbers and densities, the material of the testing containers was borosilicate glass and polystyrene material (for silver nitrate).

### Comparison of miniaturised acute *Daphnia* bioassay with the standard bioassay based on literature data

The studies listed in Table 1 used a variety of control substances to compare their datasets for large volume versus low volume daphnid tests. These assays were of course adapted for different reasons but will be used here as a standard of comparison for our own results. Hence in Table 3, we summarised the respective EC_50_ values and general conclusions that have been made by the authors on the miniaturisation approach. Overall, none of the EC_50_ values differed more than a factor of 2 between the conventional and the miniaturised tests and all authors of the studies thus assumed a good comparability of the two methodological approaches.

**Table 3.** Overview of reference chemicals that have been used to compare *Daphnia* sensitivities between test set-ups of miniaturised *Daphnia* assays and standard guideline volume test set-ups

Substance (CAS RN)	Miniaturised test EC_50_ (mg/L) (all concentrations nominal)	Standard test (mg/L) or literature data	Conclusions (copied from references)	Reference
Kepone (143-50-0)	1.6	1.6	‘Toxicity values, as well as the variation among tests, using the miniaturised test system were very similar to those values using the standard U.S. EPA methods. Therefore, it appears that the miniaturised test system can be used to conduct toxicity tests and provide accurate results.’	[14]
Linear alkyl benzene sulfonate (LAS), (-)	8.4	7.66		
Pentachlorophenol (87-86-5)	2.23	2.73		
Sodium lauryl sulfate, (151-21-3) Synthetic effluent composed of 12 chemicals (each 1 mg/L)	21.8	12.7		
Triclosan (3380-34-5), (dosing via spiking of extracts of pristine creek water with triclosan)	Modelled EC_50_ range of three independent test labs: 0.351–0.516	*Geometric mean from reported literature in DiPaolo et al.: 0.403*	‘EC_50_ values obtained with the different test set-ups in different laboratories are in good accordance, tests show comparable sensitivity.’	[21]
Acridine (260-94-6), (dosing via spiking of extracts of pristine creek water with triclosan)	Modelled EC_50_ range of four independent tests: 3–5.1	*Geometric mean from reported literature: 3.76*	See above	[21]
Cadmium chloride (10108-64-2)	0.98–1.4	1.4 1.4–1.91	‘Although from our toxicity measurements for cadmium chloride … we observe that the % mortality induced … may vary slightly across different experiments, in all cases there were no significant differences between the different conditions tested.’	[13]
Nickel chloride	9.1–14.3			
Formamide	~0.8		The same observation was made for nickel chloride and formamide.	
K_2_Cr_2_O_7_	0.518	0.557	‘The sensitivity of daphnids towards K_2_Cr_2_O_7_ was comparable (based on EC_50_ values) between test set ups.’	[15]
AgNO_3_	0.0031	Not analysed/analysable	‘Comparable AgNO_3_ toxicity was also reported by others (Allen et al. 2010; Asghari et al. 2012; Karen et al. 1999).’ References cited in [15]	[15]

### Comparison of our miniaturised acute *Daphnia* bioassay with the conventional bioassay by testing 16 selected substances

**Figure 1:**
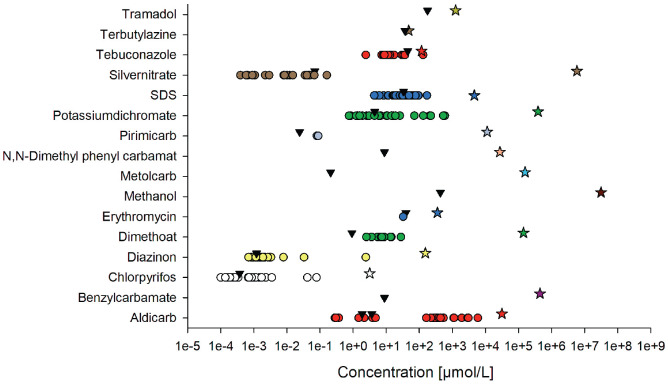
Retrieved US EPA ECOTOX EC_50_ data (acute *Daphnia* immobilisation after 48 h) of the 16 test substances in comparison to UFZ Biotox data (miniaturised approach, black triangles). In addition, the water solubility limits are shown (star symbols). Two independent miniaturised tests were done with Aldicarb (thus two triangles are depicted in the figure).

Sixteen substances with existing data for the conventional approach from the UFZ laboratory, as well as with increasing logKow were tested in the miniaturised assay to evaluate the possible differences in the sensitivities due to laboratory-specific handling, cultivation, etc. (Fig. 1 and Table 2). Problems with *Daphnia* swimming behaviour or deviation from normal behaviour due to reduction of height of the water columns was not observed. The exact physico-chemical and other information about the substances are collected in Table 2.

For the comparison of both test approaches, only EC_50_ immobilisation (48 h exposure) values were used. Parameters of all concentration–effect curves are shown in Appendix (Table A1).

Key results, also presented in Fig. 1, show a toxicity range in terms of EC_50_ for all test substances of roughly between 1 and 100 μmol/L. Three substances – chlorpyrifos, dimethoate and silver nitrate – were specifically more toxic than the rest (EC_50_ data ranging from 0.0001 to 0.001 μmol/L). The EC_50_ values of the miniaturised toxicity tests, as performed here, indicate a general trend of a slightly higher sensitivity compared to the geomean of the published EC_50_ values from the US EPA ECOTOX database (see Table 2). The negative controls did not show any difference in immobilisation between the two test approaches.

### ECOTOX database retrieval for comparing miniaturised with conventional daphnid tests

Table 2 also shows the literature data retrieved from the US EPA ECOTOX database for the 16 selected test substances (https://cfpub.epa.gov/ecotox/) (see also [28]). Data are depicted as the geometric mean of all retrieved data (see Material and methods section for exact search parameters). Figure 1 is the graphical presentation of Table 2. Key results show that lipophilicity (as logKow) ranged from −0.77 to 4.96 (MeOH and chlorpyrifos, respectively) with an equal number of substances from logKow <1–2 and >2. Baseline toxicity, as calculated with the formula published in the ECOSAR software (Version 1.11), varied over five orders of magnitude with predominance of substances with a baseline toxicity of between 0.1 and 2 mmol/L. Chlorpyrifos was the substance with highest baseline toxicity (0.0011 mmol/L) while MeOH had the lowest (588 mmol/L). Comparison of EC_50_ values retrieved from the ECOTOX database, the conventional approach (analysed in glass) and the miniaturised test (also analysed in glass) did actually show differences between the EC_50_. But these were not greater than a factor of 2–3.

Data from the ECOTOX database usually covered a range of at least three orders of magnitude. The EC_50_ of the miniaturised toxicity tests usually were within a factor of 2–3 to the geomean of the literature data with two exceptions, dimethoate and pirimicarb. Dimethoate and pirimicarb data from the miniaturised assay did show lower EC_50_ than the published ECOTOX database data (roughly a factor of 8 and 4 with dimethoate and pirimicarb, respectively – pointing to a somehow higher sensitivity). For six substances no data at all could be retrieved from the ECOTOX database. Here, no comparison with our data was possible.

## Discussion

In recent aquatic monitoring and assessments, the sample volume sizes have decreased steadily. For example, in the framework of the German National Action Plan (NAP) to evaluate the pesticide contamination of creeks close to agricultural used land (Small Stream Monitoring Project financed by the German Federal Ministry for the Environment, Nature Conservation and Nuclear Safety (BMU), research code 3717634030), https://www.ufz.de/kgm/index.php?en=44480. Within this project, the pesticide contamination of small streams in Germany was assessed with diverse methods. One assessment approach included the rain event sampling (‘agricultural run-off’) of water and the concentration, extraction and, finally, quantification of the water contaminants such as pesticides. These extracts were then supposed to be analysed via different bioassays to evaluate their ecotoxicological activity in parallel to the known pesticide contamination. As many bioassays needed to be tested, the sample volume for each bioassay was restricted. One main motivation in this special monitoring project was the need to deal with low water sample volumes (e.g., water extracts) as are used in surface-water monitoring [29,30], and still enable a reliable biological effect analysis with standard biotests. These biotests included in vitro tests with different cell lines [31] and organismic biotests such as microalgae, *Daphnia* and zebrafish embryos. In addition to monitoring of pesticide contamination, the miniaturised *Daphnia* assay might be also used for other purposes. Small volume testing could be used for leachate analysis [6], the testing of nanomaterials [15] or the analysis of microplastic effects. For all these above-mentioned purposes, only small amounts of test volume can be produced and thus used in the bioassays. The main goal of this work was to evaluate existing research data and verify a reliable *D. magna* immobilisation assay in a miniaturised format specifically for the testing of samples with limited volume. The hypothesis we followed was based on the theory that a decrease in the test volume, that is, increase of density of the acute *Daphnia* test, would not have negative effects on sensitivity. Thus, for verification, data obtained in the conventional acute *Daphnia* test with a duration of 48 h as described in OECD202 [2] as well as the miniaturised format were systematically compared for 16 selected substances (see Fig. 1). As a consequence, the data and results did not include shorter or longer exposure times than the 48 h, although in nature a pulse of toxin exposure might of course be different from the standardised 48 h exposure.

With the literature search, a rather smaller number of seven publications was found which directly had the purpose of also using a miniaturised assay in one way or another. All publications showed more or less that miniaturisation combined with a decrease in volume and increase in density of daphnids did not change the single substance EC_50_ results by usually more than a factor of 2–3 [13]. We compared a variety of different parameters for the testing and observed no significant difference in sensitivity to cadmium and nickel chloride and formamide, for example. Our adapted *Daphnia* test in 24-well glass titre plates was very much comparable to the identified publications with the one exception that most of the studies used plastic material (i.e., polystyrene) micro titre plates. In conclusion, the differences seen with the selected substances were too small to infer that the miniaturisation would completely mislead an assessment under the test conditions used. Furthermore, no obstacles regarding animal behaviour were reported. Our comparison of conventional and miniaturised toxicity values for 16 selected substances was in line with these observations. Also, data for both approaches fit into the dataset retrieved from the US EPA ECOTOX database, with EC_50_ values clearly being within the range of observed toxicity values (as shown in Fig. 1). Overall, the highest variation of published toxicity data was observed for aldicarb (5 orders of magnitude). Here it needs to be pointed out that no information on the test format was retrieved and we assume a variation of approaches was used. Beside that high range of EC_50_ only data from two different *Daphnia* species were used (*D. magna* and *Daphnia laevis*). Still, out of necessity, the hypothesis was that a) usage of different clones of the same species and b) the sensitivity of the different daphnid species would be comparable (at least for the first hypothesis [32] showed that this might not be the case). This comparability was not checked for all 16 substances though and thus it cannot be excluded, that some of the variances of the EC_50_ data observed are due to a possibly higher or lower sensitivity of the different daphnid species compared to *D. magna*. Data of the other substances (beside aldicarb) came from tests with 13 other species (*Daphnia carinata*, *D. laevis*, *Daphnia longispina*, *Daphnia ambigua*, *Daphnia pulex*, *Daphnia similis*, *Daphnia obtusa*, *Ceriodaphnia dubia*, *Ceriodaphnia reticulata*, *Ceriodaphnia rigaudi*, *Ceriodaphnia cornuta*, *Moinodaphnia macleayi*, *Moina macrocopa*). But even with this comparable high number of daphnid species, the majority of published daphnid tox data were generated with only four species (*D. magna*, *D. laevis*, *D. pulex* and *C. dubia*). Such a high variability in toxicity data might also be due to the different sensitivities of the daphnid species. In contradiction to that, a review by Wogram et al. [33] showed that *D. magna* is among the most sensitive species (referring to organic substances) and that more sensitive species do not differ from *D. magna* by more than a factor of 10. In addition, a recent literature study (by the Procter and Gamble Company together with the US EPA) did not find any differences between the species sensitivity of *D. magna* and *D. pulex* in acute and chronic tests [34]. Nevertheless, some substances did show differences of one to two orders of magnitude between the two species. Here, also a possible effect of nutrition of adult daphnids on sensitivity of the offspring might also explain some of the differences as was shown by [35] for cadmium. Neonates of well-fed adults were 2–3 times less sensitive than the less-fed adult offspring. This difference was explained by possible energy limitation for detoxification of cadmium which could also be a reason of sensitivity difference in other tests substances. Barata et al. [36] found that differences in tolerance to certain metals were influenced by water hardness among *D. magna* clones, that genetic variations influence sensitivity to toxins [37] and that phenotypic plasticity ([38] and citations therein) further increases the complexity to control sensitivity in toxicity tests, as a whole suite of parameters may ‘disturb’ a controlled experimental setup. Although the above observations by Enserink et al. [35] may only be transferred to other metals, it seems plausible that energy limitation due to detoxification could also be a factor in other toxicity tests. In addition, Olkova et al. [39,40] showed that the test water composition may also influence sensitivity of neonates.

Sixteen test substances were selected, covering a wide range of lipophilicity, to also analyse potential substance loss due to sorption processes to the walls of the glass well plate. This possible loss is also predominantly covered in the OECD standard test guidance #23 [27], ‘Testing of difficult substances’, remarking that an estimated loss of more than 20% of the starting concentration during testing should be paralleled by chemical quantification. As this is an even bigger challenge with test vials made from plastic (the most often used test vessel material), quite a few papers have covered different test systems, organisms and cell lines, and tried to pin down the various parameters which might interfere with a more realistic toxicity assessment of tests done in small volumes, especially in polystyrene titre plates. The parameters reviewed included the definition of thresholds for physico-chemical parameters such as lipophilicity and resulting sorption to test well material, sorption to test medium and other variables, for example, volatility or halflife [41–48]. Others [49] introduced passive dosing for testing hydrophobic organic substances. For example, polydimethylsiloxane (PDMS) for testing the effects of polycyclic aromatic hydrocarbons (PAH) with a logKow of above 3.5 show the better reproducibility of tests done with silicone-based materials.

The results cited above were published with the assumption that sorption of lipophilic substances to plastic-based well plate material might be substantial and may also interfere with testing even in glass if the surface to volume ratio is high. The loss of bioavailable test substances would increase the risk of underestimation of toxicity and thus mislead hazard/risk assessors. All the papers cited above gave limits, thresholds or workarounds to deal with a possible loss of the bioavailable fraction. These included logKow limits in microalgae and fish embryo testing [45,46], but also solutions for calculation or minimisation of possible loss [41,48]. To sum up, a logKow of around 3 or above may pose a risk of loss larger than 20%. So, a loss of substances due to sorption or volatilisation can be expected in the miniaturised test [45,46]. The goal of this work however, was not to show the differences between titre plate material (glass vs. plastic or open vs. closed exposure systems) but to see whether the volume decrease (i.e., density increase) might pose a risk for underestimation of toxicity. Still, in other publications, a miniaturised assay was used for risk assessment of water extracts [8,20,50,51] without any obvious problems in terms of higher negative control effects. As the quantification of substance concentration was not done by us, data were compared to literature data, in which mostly no quantification was done either [52]. Comparison was on the level of EC or LC_50_ results. Data of the miniaturised *Daphnia* assay most often were close to the mean or geometric mean of the literature data and thus seemed to be of similar quality. This is in concordance with other publications and meets our expectations of a similar sensitivity. With the 16 substances tested and the 13 which could be directly compared, no effects could be seen which might be explained by the higher density of daphnids per volume of test well. Density and also intraspecific competition is seen as being critical in sub-chronic and chronic *Daphnia* tests [53], and may have significant effects on sensitivity to toxic substances as was shown in studies by [54,55]. But with the acute tests, density did not seem to be a problem for sensitivity, at least to the chemicals tested.

## Conclusion and outlook

For *D. magna* immobilisation assays for test materials with limited sample availability, for example, water extracts, leachates and nanomaterials, there was a need to strongly reduce the volume of the medium (as in [14]). Hence, the volume of medium used per animal was reduced. The volume to neonate ratio reported in Grintzalis et al. [13] for the 24-well format using five neonates in a volume of 1.5 mL medium was adopted. The advantage is that *Daphnia* mobility and mortality are quickly and easily accessible using a microscope, because one well with five neonates can be observed at once. Furthermore, the test is more economical in terms of time for preparation, substances required and amount of toxic waste that is generated. It is anticipated to further develop this setup for a behavioural assay involving live-tracking of animals with a camera where using multi-well plates is a favourable approach [13,56]. This requires the use of one neonate per well, and hence a further reduction of volume may be anticipated.

As the testing in 24-well glass microtitre plates did not show great differences in terms of sensitivity to the substances tested in this study, it might also be useful for the analysis of nanoparticles or microplastics, or the ecotoxicological monitoring of environmental samples. The approach established here is transferable to many other types of samples with limited sample volume availability. Still, adjustments to other tests using small volumes – such as the fish embryo assay with *Daphnia rerio* embryos – might be needed as oxygen consumption or pH changes due to higher density of organisms per volume could add stress and thus distort results.

## Data Availability

The datasets generated during and/or analysed during the current study are available from the corresponding author on reasonable request.

## Appendix

**Table A1.** Concentration–effect relationships curve parameters, sigmoidal hill model, four parameters, f = y0+a*x^b/(c^b+x^b) with y0 = min, a = max, b = p = slope, c = EC_50_, of all 16 test substances

Test substances (alphabetical order)	CAS RN	Concentration–effect parameters, (24 h exposure), (min = 0, max = 100)	Concentration–effect parameters, (48 h exposure), (min = 0, max = 100)	(μg or mg/L)
Aldicarb	116-06-3	EC_50_ = 1.28 *P* = 4.50	EC_50_ = 0.36 *P* = 2.62	mg
Benzyl carbamate	621-84-1	EC_50_ = 95.31 *P* = 5.23	EC_50_ = 64.17 *P* = 2.66	mg
Chlorpyrifos	2921-88-2	EC_50_ = 1.29 *P* = 0.79	EC_50_ = 0.13 *P* = 2.02	μg
Diazinon	333-41-5	EC_50_ = 0.91 *P* = 1.78	EC_50_ = 0.37 *P* = 2.23	μg
Dimethoate	60-51-5	EC_50_ = 1.60 *P* = 1.09	EC_50_ = 0.21 *P* = 1.92	mg
Erythromycin	114-07-8	EC_50_ = 184.09 *P* = 17.86	EC_50_ = 29.05 *P* = 2.70	mg
MeOH	67-56-1	EC_50_ = 3.69 *P* = 3.81	EC_50_ = 1.76 *P* = 2.97	**%**
Metolcarb	1129-41-5	EC_50_ = 0.12 *P* = 2.75	EC_50_ = 0.034 *P* = 1.89	mg
N,N-dimethyl carbamate	6969-90-0	EC_50_ = 6.86 *P* = 2.65	EC_50_ = 1.46 *P* = 1.68	mg
Pirimicarb	23103-98-2	EC_50_ = 18.70 *P* = 3.62	EC_50_ = 5.74 *P* = 1.30	μg
Potassium dichromate	7778-50-9	EC_50_ = 1.72 *P* = 4.55	EC_50_ = 1.32 *P* = 8.61	mg
Silver nitrate	7761-88-8	EC_50_ = 17.24 *P* = 2.66	EC_50_ = 11.74 *P* = 3.56	μg
SDS	151-21-3	EC_50_ = 41.76 *P* = 19.62	EC_50_ = 9.64 *P* = 1.79	mg
Tebuconazole	107534-96-3	EC_50_ = 17.81 *P* = 153.15	EC_50_ = 13.19 *P* = 4.32	mg
Terbuthylazine	5915-41-3	EC_50_ = 18.70 *P* = 1.63	EC_50_ = 11.08 *P* = 1.79	mg
Tramadol	27203-92-5	EC_50_ = 219.99 *P* = 3.36	EC_50_ = 46.98 *P* = 1.77	mg

**Table A2.** Abstract Sifter results table (screenshot of query results of first 42 selected publications)
